# Supplementary material for: A set of multi-entry identification keys to African frugivorous flies (Diptera, Tephritidae)
Source: Zookeys. 2014 Jul 24;(428):97–108. doi: 10.3897/zookeys.428.7366 (PMC4143993; doi:10.3897/zookeys.428.7366)
Supplement: Supplementary material 6 — Key to Ceratitis [file zookeys-428-097-s006.zip › SF6_ZooKeys_key to Ceratitis/key/SF6_ZooKeys_key to Ceratitis/Media/Html/Ceratitis turneri .htm]

Ceratitis (Ceratalaspis) turneri (Munro)


***Ceratitis*** **(*Ceratalaspis*)
*turneri* (Munro)**

*Pardalaspis turneri* Munro, 1937: 5.

Body
length: 3.85 (3.75-4.25) mm; wing length: 3.49 (3.85-4.35) mm.

Male

Head.
Antenna yellow-orange except third segment brownish. Third antennal segment
twice as long as second segment. Arista with short hairs over entire length.
Face with upper half darker coloured. Frons with short scattered hairs which
are largely the same colour as frons; convex, in lateral view not distinctly
projecting forwards at antennal implant. Chaetotaxy normal for subgenus.

Thorax.
Postpronotum white, unspotted. Ground colour of mesonotum pale, partly with
orange tinge (not distinct because of broad dark bands); mesonotal pattern,
spots usually confluent forming two broad lateral stripes with white lunule,
sometimes less developed. Chaetotaxy normal for subgenus. Scapular setae black,
sometimes inner setae pale. One anepisternal bristle. Scutellum white basally,
otherwise yellow with three black separate markings apically, not extending
more than halfway scutellum (some specimens from Kenya have the spots partly
joined); basally without dark spots, only slight darker colouration.
Subscutellum completely dark.

Legs
yellow; setation typical for subgenus, mainly pale especially on femora.
Posterior and posterodorsal rows on fore femur pale.

Wing
bands with yellow areas reduced, mainly dark. Banding, marginal band with clear
interruption after cell R1; cubital and medial bands joined with marginal band;
crossvein r-m before middle of discal cell. Crossvein dm-cu posteriorly more
inwards than anteriorly. Extension cup sometimes weakly sinoid.

Abdomen.
Brownish colour. Setation and banding typical for subgenus.

Female

As
male except for the following characters: Oviscape shorter than abdominal terga
3-6 combined.

(Description
after De Meyer, 1998)
